# Experiences with Wearable Sensors in Oncology during Treatment: Lessons Learned from Feasibility Research Projects in Denmark

**DOI:** 10.3390/diagnostics14040405

**Published:** 2024-02-13

**Authors:** Helle Pappot, Emma Balch Steen-Olsen, Cecilie Holländer-Mieritz

**Affiliations:** 1 Department of Oncology, Centre for Cancer and Organ Diseases, Copenhagen University Hospital, Rigshospitalet, 2100 Copenhagen, Denmarkcecilie.hollaender-mieritz@regionh.dk (C.H.-M.); 2 Department of Clinical Medicine, Faculty of Health and Medical Sciences, University of Copenhagen, 2100 Copenhagen, Denmark; 3 Department of Oncology, Zealand University Hospital, 4700 Naestved, Denmark

**Keywords:** wearable sensors, oncology, feasibility, cancer, eHealth

## Abstract

Background: The fraction of elderly people in the population is growing, the incidence of some cancers is increasing, and the number of available cancer treatments is evolving, causing a challenge to healthcare systems. New healthcare tools are needed, and wearable sensors could partly be potential solutions. The aim of this case report is to describe the Danish research experience with wearable sensors in oncology reporting from three oncological wearable research projects. Case studies: Three planned case studies investigating the feasibility of different wearable sensor solutions during cancer treatment are presented, focusing on study design, population, device, aim, and planned outcomes. Further, two actual case studies performed are reported, focusing on patients included, data collected, results achieved, further activities planned, and strengths and limitations. Results: Only two of the three planned studies were performed. In general, patients found the technical issues of wearable sensors too challenging to deal with during cancer treatment. However, at the same time it was demonstrated that a large amount of data could be collected if the framework worked efficiently. Conclusion: Wearable sensors have the potential to help solve challenges in clinical oncology, but for successful research projects and implementation, a setup with minimal effort on the part of patients is requested.

## 1. Introduction

**Figure 1:**
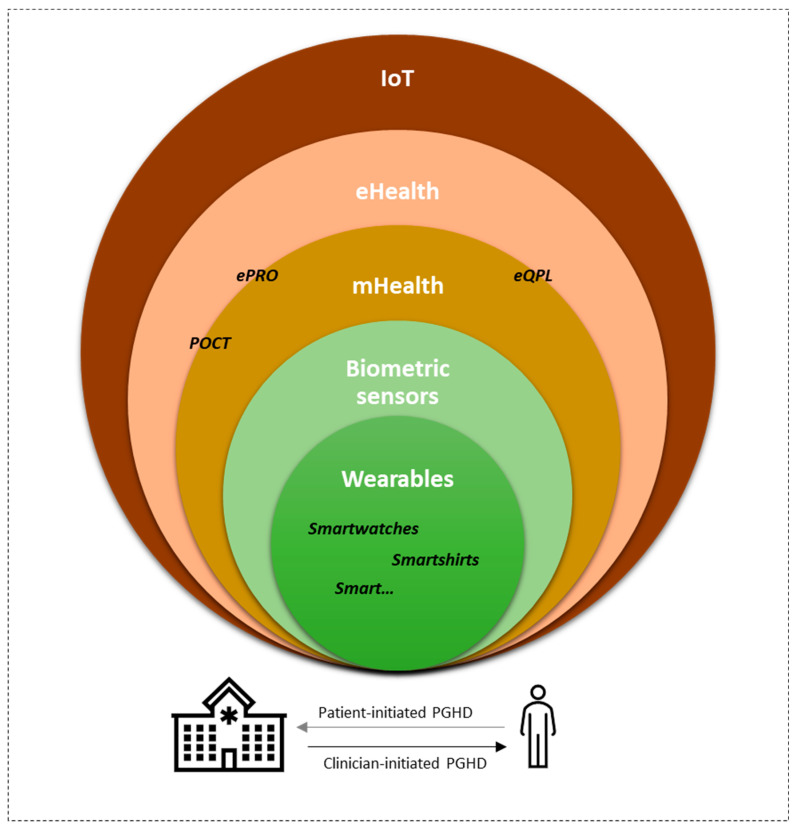
Internet of Things from a healthcare perspective. Modified from Holländer-Mieritz et al. Acta Oncol. 2020 [8]. IoT: Internet of Things; mHealth: mobileHealth; eHealth: electronicHealth; ePRO: electronic patient-reported outcome; eQPL: electronic question prompt lists; POCT: point-of-care testing; PGHD: patient-generated health data.

Oncology is facing increasing resource problems since the fraction of elderly people in society is growing, as is the incidence of some cancer diseases [1], as well as the number of treatment lines offered to each individual [2]. Hospital owners and society in general are searching for new ways of dealing with this challenging fact. Health innovations are often brought up as possible solutions [3], and new technologies such as those included in the Internet of Things (IoT) might be potential concepts to study further [4]. The IoT refers to objects that can connect to and through the internet but are dependent on human beings for information [5]. Thus, the IoT may comprise possible solutions for the increasing cancer health challenge (Figure 1). A part of IoT is eHealth, which refers to electronic health services and information [6]. eHealth has been known for the past two decades and continuously evolves [7], compromising many tested methods and emerging new technologies [8]. During the COVID-19 pandemic, it was shown that some eHealth interventions, such as phone and video consultations could be implemented relatively quickly in the hospital setting [9,10,11], and many health authorities, including the Danish health authorities, now have increased focus on innovation and research based on eHealth that can be used in the healthcare setting to increase patient quality of life while taking some of the burdens off hospitals [12,13,14]. Some eHealth solutions are already implemented in hospitals, for example, electronic health records and, to some extent, online video consultation. However, eHealth covers many possibilities at different levels, which for a great part are yet to be explored [8]. Although the health authorities see great perspectives in these new technologies within the healthcare system, there is still a long way from research to implementing the right solutions [15]. An advantage of eHealth is that present solutions house the potential to support self-management and self-empowerment [3,16,17]. Further, increased patient and public involvement (PPI) in research and clinical practice will hopefully contribute to the development and implementation of even more relevant eHealth solutions in the future [18].

eHealth solutions can also be considered as patient-generated health data (PGHD). The Office of the National Coordinator for Health and Information in the U.S. has defined PGHD as ‘health-related data created, recorded, or gathered by or from patients to help address a health concern’ [19,20]. PGHD can provide information about the patient that has not previously been available to healthcare professionals [21,22]. PGHD can either be patient initiated or clinician initiated. Patient-initiated PGHD is where the patient self-tracks their data and decides to share it with their healthcare professional. For clinician-initiated PGHD, the healthcare professional requests information from the patient [19,22]. Independent of whether PGHD are patient or clinician initiated, working with PGHD in research can be challenging due to data protection regulations, and in Europe this is regulated by the General Data Protection Regulation (GDPR), an EU regulation implemented in 2018 [23,24].

The Danish healthcare system is based on the principles of free and equal access to healthcare for all citizens, primarily financed by taxes [25]. Consequently, hospital care is free for patients with conditions that need to be treated at the hospital, such as cancer treatment. Based on this homogeneity in healthcare, Denmark might be a perfect model environment to study eHealth solutions in oncology. Although social vulnerability and inequality are also present in Denmark [26,27], the country is seen as a high-income country with good access to the healthcare system for all citizens, and Denmark is one of the countries in the world where the highest percentage of the inhabitants have access to the internet and own a mobile phone. However, challenges with digital literacy might still exist [28].

Foreseeing the upcoming resource challenges in oncology, we have performed research with clinician-initiated PGHD in a Danish oncological setting. Different PGHD tools such as wearables may be used to monitor a patient during the treatment trajectory, and the systematic use of PGHD in combination with standard care may help in the early identification and handling of side effects and, at the same time, secure a more patient-centered approach [4,29]. Our research group have experience with PGHD, including patient-reported outcomes (PRO), question prompt lists (QPL), wearables, mobile phones, and point-of-care testing (POCT, e.g., blood samples) [4,21,30]. These technologies are relevant since there is an increased focus on more treatment and supportive care in the patient’s home. At the same time, new, advanced eHealth technologies are constantly being developed [30]. eHealth technologies, such as wearables, might be potential tools to help solve the demand for more treatment and supportive care at home so that patients with less health awareness can be at home more with sufficient supportive care. It has been proposed that biometric monitoring could be used at the time of diagnosis, during treatment, and follow-up [4].

However, we request that appropriate measures, purposes, and clinical outcomes be identified before moving wearable monitoring into routine clinical care. In a review by our research group focusing on wearables during cancer treatment, we found that most studies were observational or feasibility studies in patients with breast cancer, mainly focusing on physical activity [31]. Studies have indicated that heart rate or physical activity can be affected when the patient is deteriorating [32,33,34,35]. Therefore, the purpose of using a wearable could be to monitor physical activity and help detect and counter side effects before they become severe. Wearables are sensor devices that monitor variables such as heart rate, physical activity, and skin temperature [30,36,37]. The use of wearables and monitoring of biometric sensor data in the healthcare setting could provide the hospital with parameters that would typically only be available if a patient was admitted [38]. One type of wearable is a smartwatch, commonly used as a fitness tracker. However, a smartwatch also allows for advanced integrated functions such as apps and is not only an activity tracker. Even though consumer wearables such as smartwatches have expanded early, the healthcare system’s adaptation has not developed at the same rapid pace [4,39]. For healthcare professionals to assess data from the smartwatch, a secure framework is required, and there are many software providers.

Many reports and research results have highlighted the importance of new technologies such as wearables in oncology [40,41,42,43], but most of these are focused on physical activity trackers in cancer populations with good performance [44] or in the survivorship setting [45](ref). Only a few scientific reports deal with the challenges met when applying wearables in the oncological setting. A review by Huang et al. (2022) focusing on adherence and clinical outcome in cancer trials found that most studies focused on physical activity, sleep analysis, and heart vital signs. This agrees with the systemic review by our group (2020), showing that definitions of outcome measures and adherence varied [31,46]. Taken together, how to use these wearables and their outcomes are still at an early stage. However, as with PROs, the recommendations for wearables in clinical trials are emerging. One is the clinical transformation initiative (CTTI), which has issued recommendations regarding the appropriate use of mobile technology in clinical trials [47]. Specific guidelines for designing and reporting for wearable studies in oncology will hopefully make the interpretations more conformist and clinically relevant.

This case report aims at describing the Danish research experience with wearable sensors in oncology through the insight of three planned oncological wearable research projects, including challenges and lessons learned with a special focus on adherence.

## 2. Case Presentations

To reflect the present Danish experience with the feasibility of wearable sensors in oncology, a literature search was performed. Searching the scientific database PubMed using the search term: ‘cancer and wearable sensor and Denmark and feasibility’ revealed only two studies [48,49,50](case 1 (protocol [48] and result article [49]) and 2 (protocol article [50])) and a review on the topic performed by our research group [31]; the same two studies were identified when searching ClinicalTrials.gov using the same search term. Case 3 is a study designed by the author group but not yet performed. We are unaware of any other finalized Danish studies on wearables in oncology that researched feasibility during treatment.

### 2.1. Case 1—OncoWatch1.0

#### 2.1.1. Planned Study [48]

Study design—An explorative, feasibility study investigating adherence to a smartwatch, changes in heart rate, and physical activity during radiotherapy (RT) for head and neck cancer (HNC) (ClinicalTrials.gov NCT04613232).

Population—This study was planned to include ten patients aged >18 years and was intended for primary or postoperative curative RT (5–6 fractions/week, 30–34 fractions in total) for squamous cell carcinoma of the head and neck at the Department of Oncology, Rigshospitalet, Denmark. All patients had to speak and read Danish; no severe cognitive deficits were allowed. Patients had to be included consecutively at their initial visit to the Department of Oncology.

**Figure 2:**
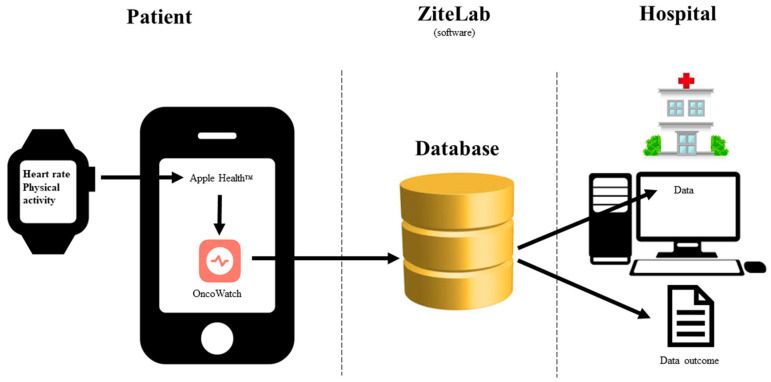
Framework for the OncoWatch1.0 feasibility study (previously published in [48]).

Device—The wearable was an Apple Watch Series 5 worn on the wrist. The watch was connected to an iPhone 8 device. The smartwatch and smartphone were supplied by the hospital. The patients had to return the devices at study termination. The patients could not use their own device, neither phone nor smartwatch. Only the patients were allowed to wear the smartwatch during the study period. The watch and phone could only be operated with a unique password. The patients were responsible for charging the watch and phone. The patients did not receive any rewards or financial support for participating in the study. ZiteLab ApS has developed the OncoWatch app, which collects data from the Apple HealthKit and sends it to a secure cloud server [51]. Data on heart rate and step count were collected. The primary investigator had access to the database. In this feasibility study, the patients were not supposed to interact with the smartwatch or the OncoWatch app. See Figure 2 for the framework setup.

Aim—This study aimed at determining the adherence to use of an Apple Watch during curatively intended RT for HNC

Research question—Can patients undergoing curatively intended RT wear a smartwatch during their treatment trajectory? We defined adherence as the number of patients who wore the device for a minimum of 12 h/day during the study period.

Planned outcomes—To determine the feasibility and adherence to the smartwatch, the primary endpoint was the number of patients who wore the device for a minimum of 12 h/day during the study period (from baseline until 2 weeks after the end of RT). Secondary endpoints were the percentage of successful data acquisition events and variations in heart rate and physical activity.

#### 2.1.2. Actual Study Performed [49]

Patients included—During the recruitment period from 22 January to 1 December 2021, 63 patients, 48 men and 15 women aged 30–82 years, were screened for enrollment in the OncoWatch 1.0 study. Twenty-seven of the sixty-three patients were never asked due to either the healthcare professionals’ assessment (*n* = 3), no reason for missing information (*n* = 11), or competing protocols (*n* = 13). A total of 23/63 patients declined participation, primarily with the reason that they could not oversee participation. Three of the sixty-three patients had disseminated diseases that had not been identified initially.

Data collected—Wear times of >12 h/d during the study period varied between 0 and 55 days, with a median of 5 days. Days with any data registration varied between 4 and 59 (33.5).

The total data acquisition rate over the study period was 61%.

The entire data file of the study provided 196,389 data points on 10 patients. Further information on duration and frequency of data collection can be found in reference [49].

Results achieved—Only two patients had a wear time of >12 h/d for 90% of the study period. The total adherence rate for wearing the watches for >12 h/d over the study period was 31%. With respect to biometric data outcome, the entire data file of the study provided 196,389 data points on 10 patients. It was preplanned that data on heart rate and physical activity (step count) would be analyzed. Since this was an explorative endpoint and due to low adherence, only descriptive heart rate and step count statistics were analyzed for the four patients with the best compliance. The results for the four patients varied and showed mixed signals, and should only be viewed as display examples; these analyses have been published elsewhere [49].

Further planned activities—It was already planned at the initiation of OncoWatch1.0 study to design a larger explorative pilot study investigating monitoring of heart rate and physical activity during radiotherapy for head and neck cancer in combination with ePRO (see case 3).

Strength/limitations—The strengths of the study were a very well-defined framework (Figure 2) and the performance of the research project in a homogenous healthcare system with equal access to healthcare services. It was a great limitation that four patients had their last data registration before their treatment course ended. These patients had experienced technical difficulties or felt it was too demanding to charge and wear the smartwatch. Further, due to the low adherence, descriptive statistics for heart rate and step count were only analyzed for the four patients with the best compliance to explore the feasibility of defining and interpreting the outcome data, and mixed signals were observed across the four patients [49]. Taken together, the outcome of the study was negative.

### 2.2. Case 2—OncoSmartShirt

#### 2.2.1. Planned Study Design [50]

Study design—An explorative study investigating the feasibility of using the Chronolife smart t-shirt during cancer treatment.

Population—The study was planned to include a total of 20 Danish patients ≥18 years old with cancer and in antineoplastic treatment. Patients were recruited continuously. Of these 20 patients with cancer, 10 (50%) <39 years old, defined as adolescent and young adults (AYA), and 10 (50%) >65 years old, defined as elderly, should be included.

**Figure 3:**
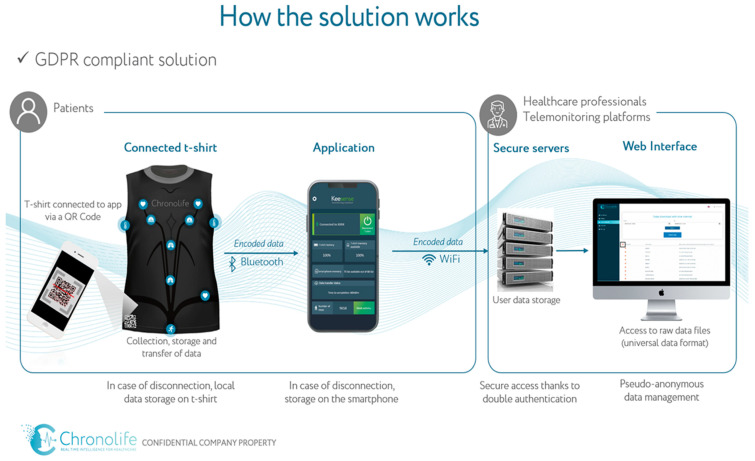
Framework for OncoSmartShirt (previously published in [50]).

Device—The study device in the trial consisted of four units; a washable smart t-shirt fitted with multiple sensors and electrodes from Chronolife, a companion smartphone or tablet app, an accredited secure data-hosting server, and a web interface [52] (see Figure 3 for the study framework). The Chronolife smart t-shirt is commercialized and “CE marked” for the consumer market. The smart t-shirt is designed for everyday use. The electrical sensors embedded in the shirt allow for the detection of six physiological parameters: ECG (beat per minute), thoracic and abdominal respiration (respiration per minute), thoracic impedance (kOhm), physical activity (steps), and skin temperature (°C) [52].

Aim—To evaluate the feasibility of using a smart t-shirt for remote monitoring of biometric sensor data in adolescent and young adults (AYA) and elderly patients during cancer treatment.

Research question—Can AYA and elderly patients wear a smart t-shirt during cancer treatment? Feasibility was measured as completion rate, defined as the number of included patients using the smart t-shirt for at least 8 h per day during the 2-week study period.

Planned outcomes—The primary endpoint was to assess the feasibility of using the Chronolife smart t-shirt in two different age groups based on completion rate, which in this trial was defined as the number of included patients using the smart t-shirt at least 8 h per day during the 2-week study period. Secondary endpoints were to assess the technical feasibility in a Danish healthcare system, including data acquisition rate and data completeness. Qualitative interviews with the patients regarding the use of the smart t-shirts were performed. Patients were asked to complete a questionnaire concerning their experience with wearing the smart t-shirt. Explorative endpoint changes in heart rate, skin temperature, physical activity, and respiration frequency could be presented descriptively.

#### 2.2.2. Actual Study Performed

Patients included—We included 10 patients (5 females and 5 males) aged 22 to 30 years, median age 27 years.

Data collected—Wear time >8 h/d during the study period varied between 0 and 6 days, with a mean of 2 days. Only three patients managed to have a mean wear time >8 h/d during their days with data registration. Days with any data registration varied between 0 and 10, with a mean of 6.4 days. Heart rate was calculated on an ECG segment considered to be reliable by the manufacture’s data-cleaning algorithm. The data quality was defined as the following ratio: Length of session with heart rate values available/Total length of the session. The mean data quality in this study from patients with data collection varied between 62.5 and 91.5%, mean of 76.5%. In this trial, the data quality value was based on the quality of the heart rate values. The thematic analysis was based on the telephone interviews. E.g., One of the quotes from the interviews described technical challenges: “You have no surplus to it at all, and then you almost need someone who can do it for you, because you can’t overcome it, you just haven’t the energy to set it up […] and you both have to remember to have the smartphone for charging, and you also have to charge the t-shirt at the same time […] and the smartphone has lost its battery, and the t-shirt has gone out too, and I can’t bear it at all, I can’t overcome it at all”.

Results achieved—This study showed that it was not possible for AYA cancer patients to wear a smart-shirt for the planned period. No one managed to wear the shirt for 8 h/d for 14 days straight. Four patients had no data registrations at all: for three of the patients, the connection between smart t-shirt and smartphone application was not successful because of technical issues or malfunctions, and for the last patient the connection was successful, but the patient did not use the smart t-shirt at all, due to disease-related issues. Further information on the duration and frequency of data collection can be found in reference [53]. The interviews revealed three main themes: (1) the t-shirt is cool but does not fit cancer patients; (2) the technique limits the use of the t-shirt; and (3) monitoring of data increase the feeling of safety. In relation to the themes, the patients experienced, e.g., that it was not possible to wear a bra under the t-shirt and that the tight fitting felt uncomfortable; further, the many technical challenges with charging and updates felt like a barrier to wearing the shirt; and lastly, the participants would have liked if HCPs had surveilled their data during the trial and intervened if they observed deteriorations in body functions.

Further planned activities—Based on the fact that none of the included AYAs with cancer were able to fulfill the planned wear time in combination with the qualitative data, suggesting that the technique limits the use of the t-shirt, it was decided to not go on with the part of the study including the elderly population, but await further development of the product before more studies with smart-shirts in this population should be considered.

Strength/limitations—A strength of this study was a very well-defined study framework; however, it became a limitation that the product was not designed for our patient population and was specifically unsuited for patients undergoing cancer treatment. A major hurdle for the patients was the technical challenges, which limited use significantly.

### 2.3. Case 3—OncoWatch2.0

#### 2.3.1. Planned Study Design

Study design—Explorative pilot study investigating monitoring of heart rate and physical activity during radiotherapy in combination with electronic PRO (ePRO).

Sequential development, including four-times inclusion of 25 patients, with each phase used to improve the next phase.

Population—100 Danish patients ≥18 years planned to undergo concomitant curative radiotherapy including lung cancer, head and neck cancer, and esophagus cancer at Rigshospitalet, Department of Oncology, Denmark.

Device—Apple Watch, ePRO app on smartwatch, mobile phone. The framework developed for OncoWatch1.0 including the OncoWatch App was planned to be reused in this project with the development of the OncoWatch App, not only to gather activity data but also to display ePRO questionnaires on the Apple Watch. This app should include feedback to patients on answers to ePRO questionnaires, nudging the patients to contact the department of oncology at severe symptom deterioration.

Aim—To investigate the use of smartwatches as an add on to symptom monitoring during high-intensity radiotherapy.

Research question—Can sensor data from smartwatches reveal symptoms of dehydration? Explorative data analysis was planned to investigate possible correlations between sensor data and clinical observations/PRO.

Planned outcomes—Correlation between sensor data and PRO data. Correlation between sensor data and dehydration. Patient feedback.

#### 2.3.2. Actual Study Performed

This study has not been initiated due to experiences from case 1 and 2. Further, another study performed by our group, the DAHANCA-38 study, focusing on ePRO for symptom-management during curatively intended radiotherapy in head and neck cancer has recruited slower than anticipated [54]. Currently, the study group has decided to await: (1) further development of devices for medical use, (2) integration of ePRO in existing electronic patient journals, and (3) possibility to use patients’ own mobile phones for data-collection. If these matters are solved, the study group will reconsider initiation of the study.

## 3. Results from the Three Case Report Studies

The aim of the present study was to describe the Danish research experience with wearable sensors in oncology through the insight of the three reported oncological wearable research projects. This included the report of challenges and lessons learned with a special focus on adherence. However, each study had other specific aims and research questions with attached results. For clarification, the results from the three studies are listed here. Further details on the design, methods, and analysis are previously reported [48,49,50,53].

- OncoWatch1.0
  Patients undergoing curatively intended RT were not able to wear a smartwatch during their treatment trajectory when successful adherence was described as wearing the device for a minimum 12 h/day during the study period [49].
- OncoSmartShirt
  AYA patients were not able to wear a smart t-shirt during cancer treatment when a successful completion rate was defined as using the smart t-shirt for at least 8 h per day during the 2-week study period [53].
- OncoWatch2.0
  The study was never performed due to technical challenges, and it was not possible to investigate if sensor data from smartwatches can reveal symptoms or signs of dehydration.

## 4. Discussion

Few studies have investigated the use of wearables during cancer treatment in oncology with a focus on adherence. We share here our experience from three planned Danish feasibility research projects on wearable sensors in oncology, all designed to take place during cancer treatment. In these studies, our major lessons were that patients found the technical issues too challenging to deal with during cancer treatment. However, at the same time, it was demonstrated that a large amount of, e.g., activity data could be collected if the framework worked efficiently. Our studies did not, however, investigate the impact of any of these data on cancer trajectory.

Research in eHealth is continuously emerging, e.g., due to the development of new device technologies. In Table 1, it can be seen how the advancement in integrating different components in our case studies has changed over time. Some of the problems we are struggling with in present studies using wearables in oncology might be eliminated in the future since new technologies point to the fact that wearables could be replaced by implantable flexible sensors for in vivo health monitoring, which offers more accurate and reliable vital sign information [55]. In the coming years, the area of wearable biosensors will probably be subject to scientific interest; however, the integration of biosensors into/with the human body, such as patches, gloves, or tattoos, might be more acceptable by the patients but even more challenging to work with in research projects than the wearables presented in our studies. Presently, we must learn from existing results and technology accessibility. Chow et al. have reported on how many of the studies reported in oncology using wearables focused on rehabilitation and often ended with concluding that in investigations, the live-time monitoring of collected data is missing [56], as is also the case for our studies. However, the research field of eHealth in oncology still seems to be at an early stage, even in some of the high-incidence diseases such as breast cancer. When Flaucher et al. very recently reviewed the literature focusing on mHealth in breast cancer care, only a few studies used objective measures such as activity sensors, and most studies were conducted on ePRO [57]. Also, an attempt to investigate the association between wearable device metrics and clinical outcomes in oncology by performing a systematic review with evidence synthesis and meta-analysis had to conclude that heterogeneity between studies hampered the comparison of results and the quantification of associations between metrics and outcomes [58]. Taken together, there seems to lack guidance on how to perform studies with wearables to obtain clinical important data, which supports our decision to postpone the study designed in case 3.

**Figure 4:**
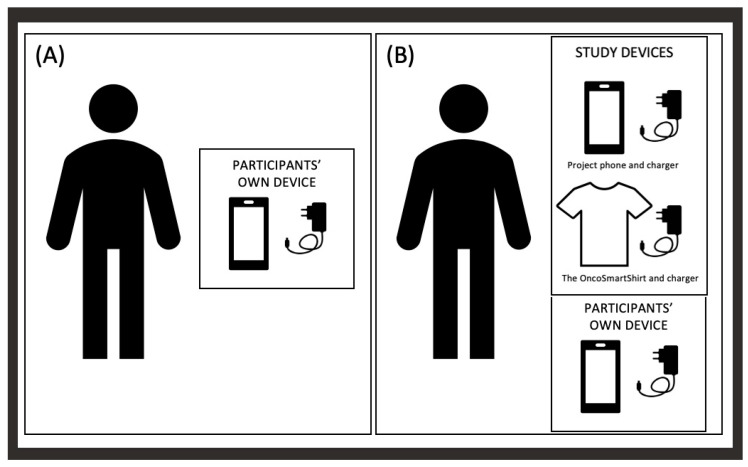
Devices acquired in ‘bring your own device’ setup (**A**) compared to research setup in public healthcare in Denmark for case study 2 (**B**).

In case study 1, a reason for low adherence was that patients had experienced technical difficulties or felt it was too demanding to charge and wear the smartwatch; and in case study 2, the qualitative data showed that the technique limited the use of the smart-shirt. This indicates that a fully decentralized ‘bring your own device’ (BYOD) smartphone-study in a population of cancer patients showed that feasibility data could be obtained from 90%. However, only 54 patients and their relatives were included [59]. The advantage of performing BYOD studies might be the familiarity with the device and the less complicated setup, ensuring fewer devices for charging and data transfer. Figure 4 illustrates the difference between daily life for a patient with a private mobile phone and a patient with a private mobile phone and participating in case study 2 concerning the number of devices and chargers, understanding the wish to perform eHealth studies using BYOD. However, caution in performing BYOD studies in research has been presented since the setup can cause imbalance [60].

Both case studies 1 and 2 showed major problems with data collection and quality, although well-defined and well-described frameworks were available. The reasons for this weakness in these studies may be multiple, and we have already pointed out the complexity of managing multiple devices and chargers at the same time as being life-threateningly ill from a severe cancer disease and receiving burdensome cancer treatment. Others have also identified challenges concerning the data quality in wearable studies. A review has identified three high-level factors that affect data quality: (1) device- and technical-related factors, (2) user-related factors, and (3) data governance-related factors [61]. All these factors have also been challenging in the Danish wearable studies in oncology and have led to inconclusive studies. We believe that the major factor that affected adherence and data collection in our studies was device- and technical-related factors, and this is an issue which may be overcome by using even smarter wearables, which are less challenging to the user. In short, this could, from a user perspective, be described as simplicity with respect to use but would technically improve devices.

To our knowledge, case study 2 was the first study to investigate the potential of a smart-shirt in an oncological setting [50] and, unfortunately, case study 3 was not realized; however, concerning case study 1, the OncoWatch1.0 study comparison with other specific findings from other studies was possible. The OncoWatch 1.0 study investigated the use of a smartwatch for collecting heart rate and physical activity information from patients with HNC receiving curatively intended radiotherapy. Although the primary endpoint adherence, defined as a wear time of over 12 h per day in the study period, was not reached, the study brought new knowledge on collecting objective data with a smartwatch in a secure setup in a public healthcare system. In this study, adherence was initially chosen as the primary endpoint based on our findings in a systematic review investigating the use of wearables in clinical trials during cancer treatment [31], where we found that the adherence rate to a wearable during cancer treatment across cancer types varied from 60 to 100%. However, as Kos et al. [58] found, we found heterogeneity in the definitions of outcome measures and adherence across studies. Further, the diagnosis groups in the review primarily had breast cancer, followed by gastrointestinal and lung cancer. None of the studies had HNC as their primary diagnosis group, and this patient group generally appears to be underrepresented in these studies [31,46]. Basing our outcome choice on studies from other disease populations could have been unrealistic, since HNC patients are known to be socioeconomic challenged [62]. In the OncoWatch 1.0 study, we wanted to test the feasibility of a smartwatch and the setup of data transfer. We chose a smartwatch instead of a simpler activity tracker as they have more functionalities, including notifications, updates, and interactive use. This type of wearable is becoming more common among the public. We hypothesized that the integrated functions for collecting health data could play a role in symptom management in the long term. In addition, a smartwatch could allow for integrating PROs in the watch or in an app on a connected phone, which we planned to use in case study 3. The adherence rate (wear time >12 h/d in the study period) of 31% was, however, much lower than we had anticipated.

It was an advantage that the chosen setup allowed all patients to participate despite their socioeconomic situation, since the hospital delivered the smartphone and smartwatch. However, a design with both options, BYOD or supplied by the hospital, could be a better solution. Disappointingly, the data outcome in the OncoWatch1.0 study could only be used as a descriptive display due to the low adherence rate. Sher et al. (2022) [63] showed in a pilot study that patients with HNC with a decrease in step count the previous week had a significant decrease in quality of life, but this study could also not report on their primary endpoint compliance (wear time) because of technical issues. Although, to some extent, the results from this and our two other case studies of feasibility of wearables during cancer treatment are disappointing, we, as others, advocate that wearables hold a potential for future cancer care. Other researchers have mentioned that another aspect of using sensor data collected by patients is the empowerment it can add when patients are responsible and have access to the data [16,64]. Participants in the OncoSmartShirt study also mentioned that data monitoring increased the feeling of safety.

The present studies all lack data collection allowing for the use of advanced statistic models. The missing data are due to very low adherence in both study 1 and 2. But, it seems evident that wearables such as the smart t-shirt described in study 2, having six embedded sensors with continuous measurement of physiological functions [52], have the potential to generate large amounts of data suitable for using machine learning to detect patterns relating to clinically important events as well as the further development of algorithms which can be used to improve clinical care. However, if such smart technologies are taken into use in clinical practice, it is important to use formal verification and validation techniques for improving quality and ensuring the correctness of IoT services [65].

## 5. Conclusions

All three case studies on wearables intended to be used in oncology during cancer treatment revealed new and important perspectives and knowledge, which point to why achieving high compliance in wearable research in the oncological setting can be difficult. They also emphasize that wearables have potential, but one of the challenges is the research framework compared to more straightforward technical solutions or BYOD. If successful research studies are conducted and solutions are implemented, a setup with minimal effort is requested for patients, including more innovative devices. However, we advocate that patients are ready to integrate the IoT into healthcare to help solve the growing healthcare burden and provide the best possible care during their cancer trajectory.

## Figures and Tables

**Table 1:** Advancement in feasibility studies of wearables in oncology in Denmark.

	Device	PRO	User Interviews	Own Device	Feed-Back to Patient	Surveillance in Study
OncoSmartWatch1.0	Apple Watch	-	-	-	-	-
OncoSmartShirt	Chronolife t-shirt	-	+	-	-	-
OncoSmartWatch2.0	Apple Watch + ePRO	+	+	-	+	-

+/- refers to if specific functionalities/activities were included or not in the specific feasibility study.

## Data Availability

No new data were created for this case report. For access to data from feasibility studies, this has previously been reported [49,53].
